# Supplementary material for: Glutathione triggers leaf-to-leaf, calcium-based plant defense signaling
Source: Nat Commun. 2025 Feb 24;16:1915. doi: 10.1038/s41467-025-57239-1 (PMC11850895; doi:10.1038/s41467-025-57239-1)
Supplement: Supplementary file 1 — Supplementary Information [file 41467_2025_57239_MOESM1_ESM.pdf]

Supplementary Information

Glutathione triggers leaf-to-leaf, calcium-based plant defense signaling

Rui Li[1,2]†, Yongfang Yang[1,3]†, Hao Lou[1], Weicheng Wang[1], Ran Du[2], Haidong Chen[2], Xiaoxi Du[1,4], Shuai Hu[1,4], Guo-Liang Wang[5]*, Jianbin Yan[2]*, Xiaoyi Shan[1]*, Daoxin Xie[1,6]*

Affiliations:

[1]MOE Key Laboratory of Bioinformatics, Tsinghua-Peking Joint Center for Life Sciences, and School of Life Sciences, Tsinghua University, Beijing 100084, China.

[2]Shenzhen Branch, Guangdong Laboratory of Lingnan Modern Agriculture, Key Laboratory of Synthetic Biology, Ministry of Agriculture and Rural Affairs, Agricultural Genomics Institute at Shenzhen, Chinese Academy of Agricultural Sciences, Shenzhen, China.

[3]Key Laboratory of Seed Innovation, Institute of Genetics and Developmental Biology, Chinese Academy of Sciences, Beijing, 100101, China.

[4]Hainan Key Laboratory for Biosafety Monitoring and Molecular Breeding in Off-Season Reproduction Regions, Institute of Tropical Bioscience and Biotechnology & San Ya Research Institute, Chinese Academy of Tropical Agricultural Sciences, Haikou, China.

[5]Department of Plant Pathology, Ohio State University, Columbus, OH 43210, USA.

[6]State Key Laboratory of Hybrid Rice, Hunan Hybrid Rice Research Center, Hunan Academy of Agricultural Sciences, Changsha, 410125, China.

*Corresponding author. Email: daoxinlab@mail.tsinghua.edu.cn (D. X.); shanxy80@mail.tsinghua.edu.cn (X.S.); jianbinlab@caas.cn (J.Y.); wang.620@osu.edu (G.-L.W.);

†These authors contributed equally to this work.

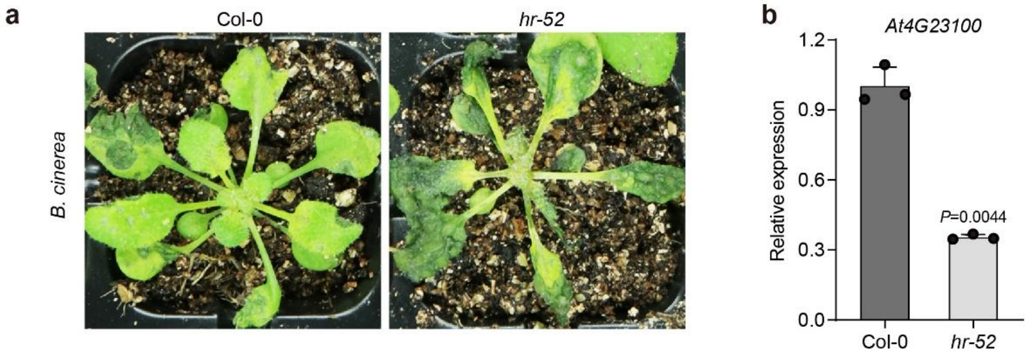

Supplementary Figure 1. Identification of *AT4G23100* by genetic screening of the *Arabidopsis* transgenic RNAi library. a Genetic screening of ~20000 primary transgenic plants identified a hairpin RNA transgenic line, *hr-52*. *hr-52* exhibited obvious susceptibility to *B. cinerea* infection. **b** Quantitative expression of *AT4G23100* in *hr-52* and Col-0 plants. Data are mean ± SD (n = 3). Statistical significance was determined by two-sided Welch's t test. Source data are provided as a Source Data file.

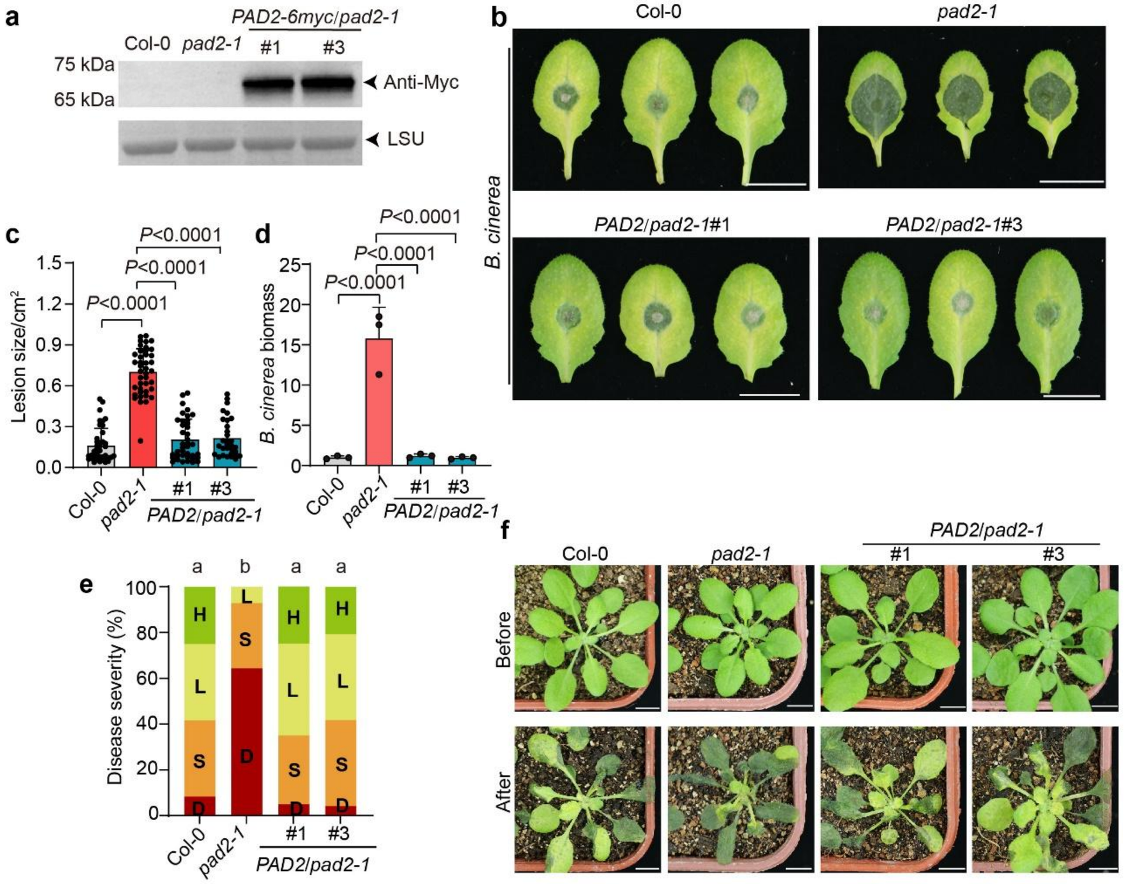

Supplementary Figure 2. ***pad2-1* is more susceptible to *B. cinerea* infection. a** Western blot analysis of PAD2-6myc protein expression in *PAD2*/*pad2-1* transgenic plants. LSU (Large Subunit of Rubisco protein) serves as a loading control. **b** Representative phenotypes of infected leaves 2 days after *B. cinerea* infection. Scale bars: 1 cm. **c, d** Quantification data of lesion sizes **(c)** and *B. cinerea* biomass **(d)** in detached leaves 2 days after *B. cinerea* infection are shown. Data are mean ± SD (n = 41 for Col-0, n = 41 for *pad2-1,* n = 39 for *PAD2*/*pad2-1*#1, n = 31 for *PAD2*/*pad2-1*#3) for (c). Data are mean ± SD (n = 3) for (d). Statistical significance was determined using one-way ANOVA followed by post-hoc Dunnett's test. **e** Plant disease severity of *B. cinerea*-sprayed soil-grown seedlings. Disease severity rating is represented as healthy (H, green), light symptoms (L, pale green), severe symptoms (S, orange-yellow), or completely dead plants (D, red). Different letters indicate significant differences ($P < 0.05$), determined with Kruskal-Wallis test. **f** Disease symptoms of 4-week-old Col-0, *pad2-1*, *PAD2/pad2-1*#1 and #3 plants

before and after spray inoculation with *B. cinerea* for 3 days. Scale bars: 1 cm. Source data are provided as a Source Data file.

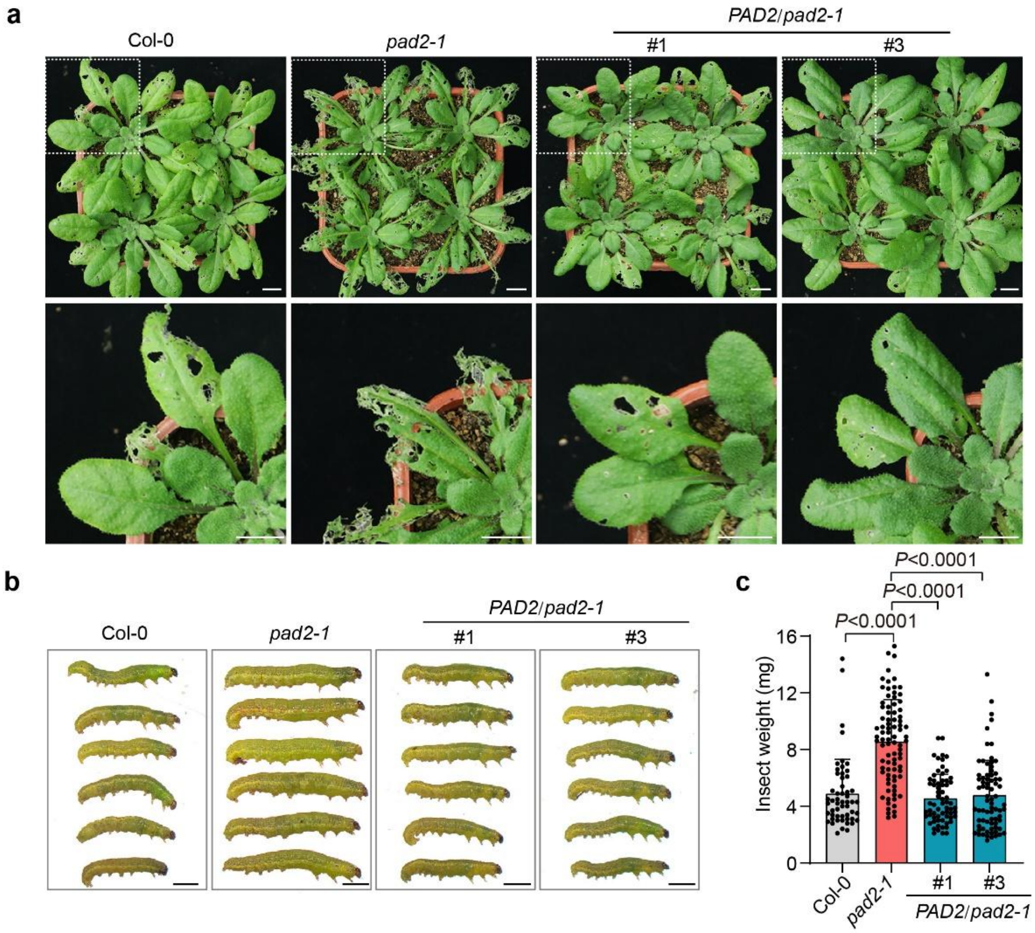

Supplementary Figure 3. *pad2-1* is more susceptible to *S. exigua* feeding. a The photographs of Col-0, *pad2-1*, *PAD2*/*pad2-1*#1 and *PAD2*/*pad2-1*#3 plants after 7 days of feeding with *S. exigua*. The bottom photographs are enlargements of some chewed leaves. Scale bars: 1 cm. **b, c** The representative images of *S. exigua* larvae **(b)** and quantification data of *S. exigua* larvae weight **(c)** recovered from Col-0, *pad2-1*, *PAD2*/*pad2-1*#1 and *PAD2*/*pad2-1*#3 plants after 7 days of feeding. Data are mean ± SD (n = 54 for Col-0, n = 85 for *pad2-1,* n = 66 for *PAD2*/*pad2-1*#1, n = 75 for *PAD2*/*pad2-1*#3). Statistical significance was determined using one-way ANOVA followed by post-hoc Dunnett's test. Scale bars: 2 mm. Source data are provided as a Source Data file.

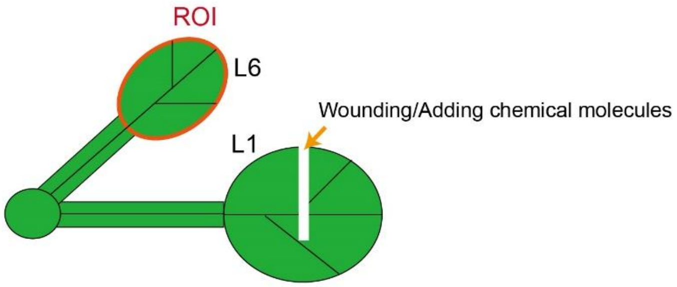

Supplementary Figure 4. Diagram showing the regions of interest (ROI) used to assess the $[Ca^{2+}]_{cyt}$ increase in systemic leaf 6 after wounding or application of chemical molecules to leaf 1. The entire leaf 6 was used to analyze the $[Ca^{2+}]_{cyt}$ increase.

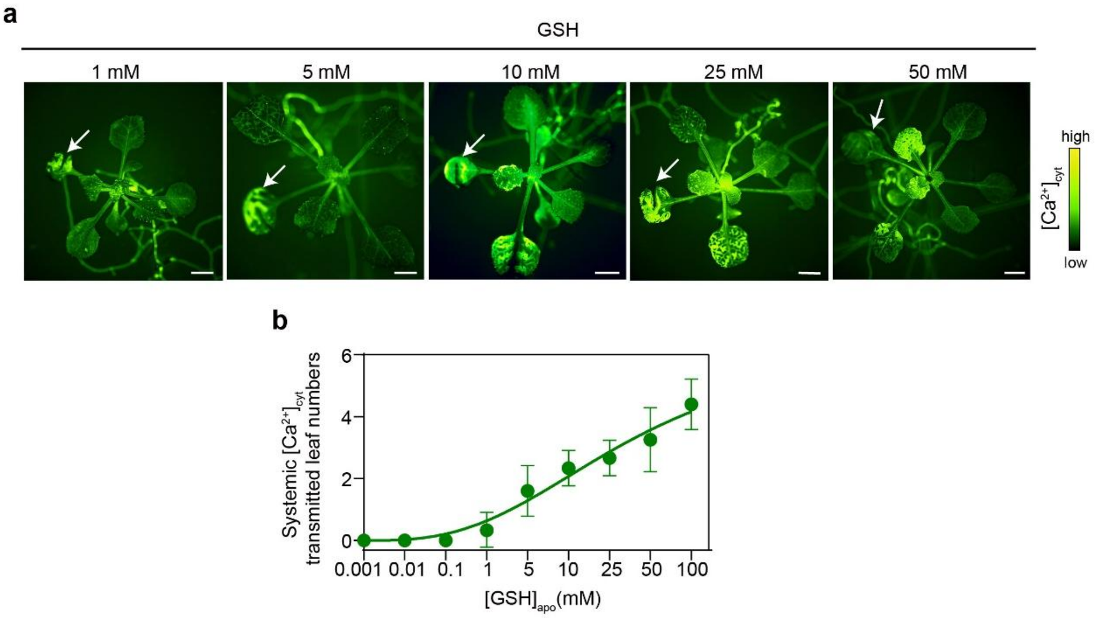

Supplementary Figure 5. GSH triggers a long-distance transmission of $[Ca^{2+}]_{cyt}$ increases in a concentration-dependent manner. $[Ca^{2+}]_{cyt}$ fluorescence signal imaging **(a)** and numbers of systemic leaves with apparent $[Ca^{2+}]_{cyt}$ transmission **(b)** after application of GSH with indicated concentrations to the leaf (white arrow) of *GCaMP3* plants are shown. Data are mean ± SD (n = 3 for GSH concentrations of 0.001 mM, 0.01 mM, 0.1 mM, 1 mM, 10 mM, and 25 mM; n = 6 for 5 mM and 100 mM GSH; n = 8 for 50 mM GSH). Scale bars: 2 mm. Source data are provided as a Source Data file.

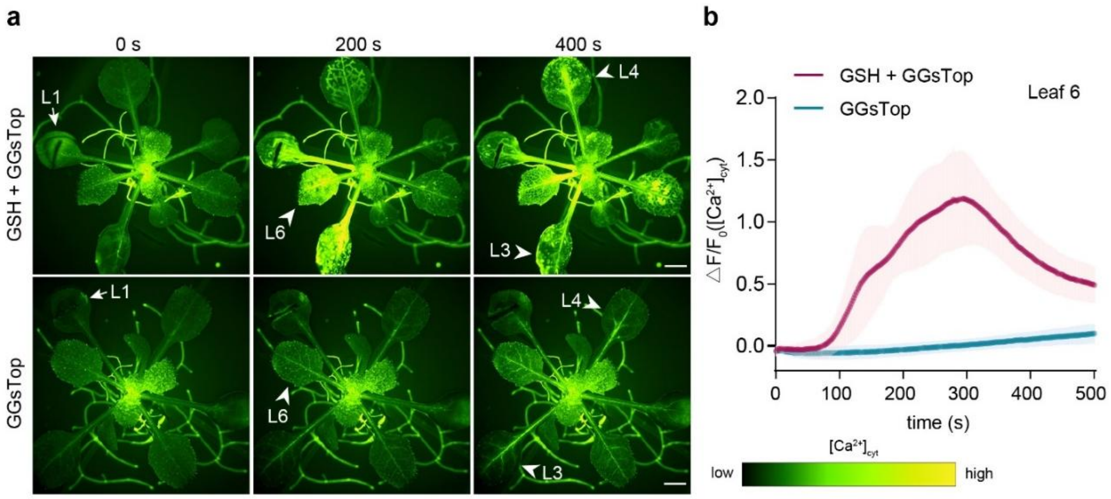

Supplementary Figure 6. Application of GGsTOP fails to suppress GSH-triggered systemic $[Ca^{2+}]_{cyt}$ transmission in plant. a $[Ca^{2+}]_{cyt}$ fluorescence signal imaging of *GCaMP3* plants after application of 100 mM GSH combined with 0.5 mM GGsTOP and 0.5 mM GGsTOP alone to leaf 1 (L1) (white arrow, 0 s) respectively. White arrowheads (200 s, 400 s) indicate leaf 6 (L6), leaf 3 (L3) and leaf 4 (L4). Scale bars: 2 mm. **b** Quantitative measurement of $[Ca^{2+}]_{cyt}$ levels in target leaf 6 at indicated times after application of 100 mM GSH combined with 0.5 mM GGsTOP and 0.5 mM GGsTOP alone to leaf 1 of *GCaMP3* plants. Data are mean ± SD (n = 12 for GSH + GGsTOP, n = 7 for GGsTOP). Source data are provided as a Source Data file.

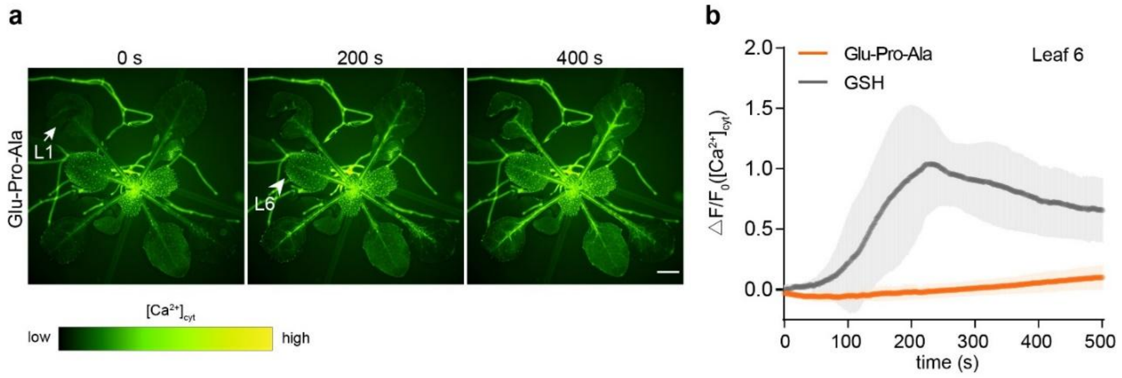

Supplementary Figure 7. Application of Glu-Pro-Ala to leaf 1 failed to induce long-distance $[Ca^{2+}]_{cyt}$ transmission in *GCaMP3* plants. a $[Ca^{2+}]_{cyt}$ fluorescence signal imaging of *GCaMP3* plants after 100 mM Glu-Pro-Ala application (white arrow, 0 s) to leaf 1 (L1). White arrowhead (200 s) indicates leaf 6 (L6). Scale bar: 2 mm. **b** Quantitative measurement of $[Ca^{2+}]_{cyt}$ levels in target leaf 6 at indicated times after application of 100 mM Glu-Pro-Ala to leaf 1 of *GCaMP3* plants. Data are mean ± SD (n = 9). GSH-induced *GCaMP3* data from Fig. 2c are reproduced (gray lines) to aid in comparison. Source data are provided as a Source Data file.

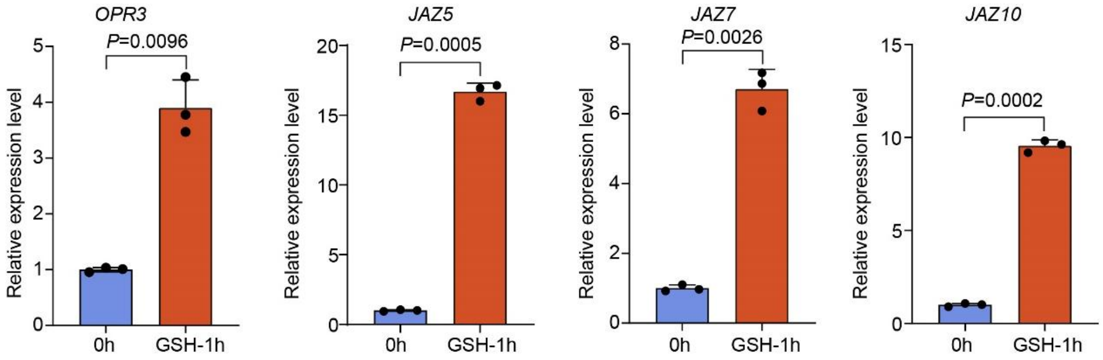

Supplementary Figure 8. Relative gene expression level of JA-responsive genes in the target leaf 6 one hour after application of 100 mM GSH to leaf 1 of WT plants. Data are mean ± SD (n =3). Statistical significance was determined by two-sided Welch's t test. Source data are provided as a Source Data file.

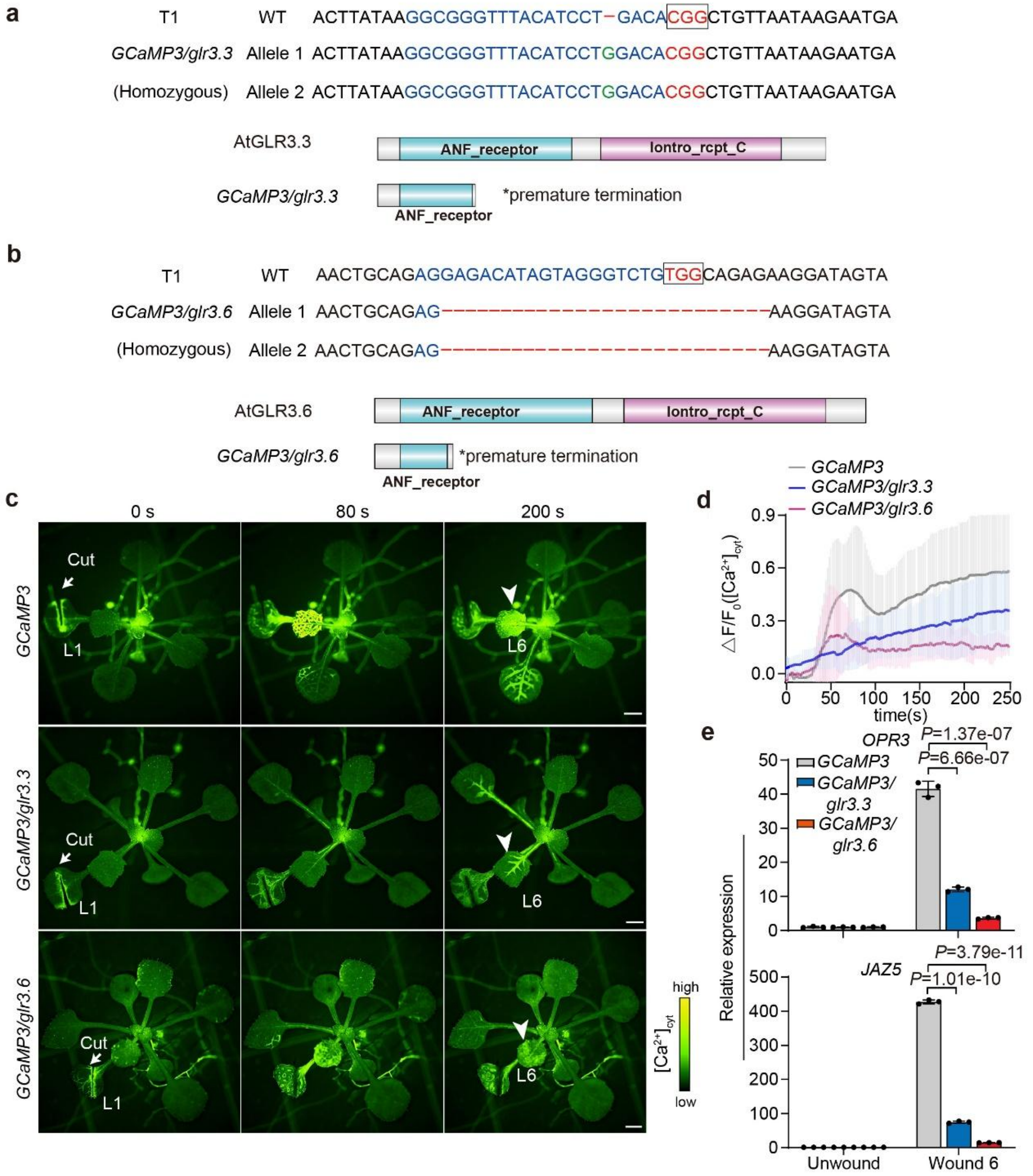

Supplementary Figure 9. Genome editing of *glr3.3, glr3.6* single mutant in *GCaMP3*. **a, b** Mutant alleles and predicted protein translation of genome-edited genes identified in regenerated *GCaMP3/glr3.3* **(a)** and *GCaMP3/glr3.6* **(b)** mutants. Blue letters indicate the target site, red letters in the small rectangular frames indicate the protospacer adjacent motif, minus symbols represent deletions, and green letters represent base insertion. **c-e** Wound-induced systemic $[Ca^{2+}]_{cyt}$ and plant defense responses are reduced in *GCaMP3/glr3.3* and *GCaMP3/glr3.6* mutants. The representative images of $[Ca^{2+}]_{cyt}$ fluorescence signal in

whole seeding **(c)**, quantitative measurement of $[Ca^{2+}]_{cyt}$ levels **(d)** and defense gene expression levels **(e)** in target leaf 6 (L6) (white arrowhead, 200 s) at indicated times after wounding leaf 1 (L1) (white arrow, 0 s) of *GCaMP3*, *GCaMP3*/*glr3.3* and *GCaMP3*/*glr3.6* plants. *GCaMP3* data from Fig. 1g are reproduced (gray lines) to aid in comparison. Data are mean ± SD (n = 6, 6, 14 for *GCaMP3*/*glr3.3*, *GCaMP3*/*glr3.6* and *GCaMP3* in d, n = 3 for e). Statistical significance was determined by Dunnett's test. Scale bars: 2 mm. Source data are provided as a Source Data file.

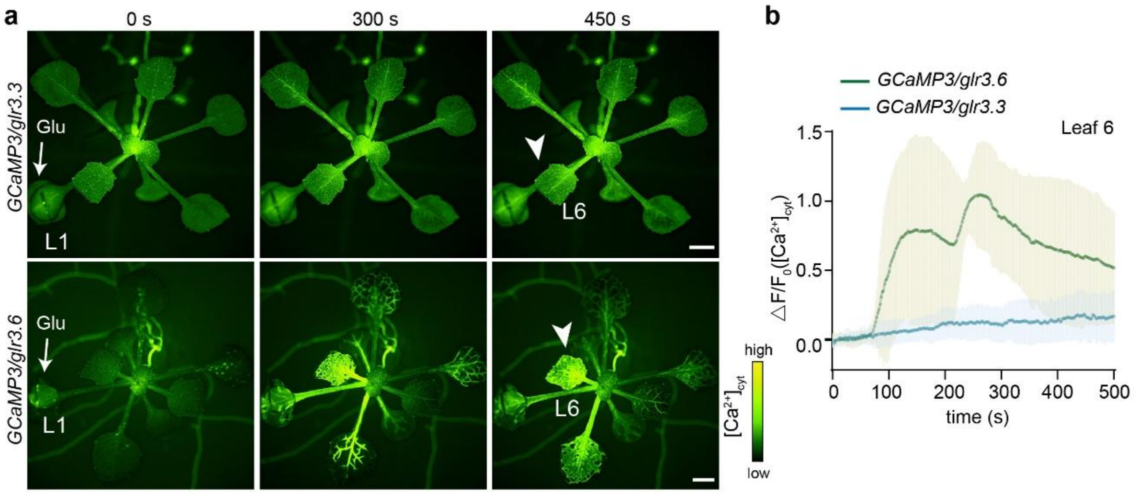

Supplementary Figure 10. Glu-triggered long-distance $[Ca^{2+}]_{cyt}$ propagation was dependent on GLR3.3. The representative images of $[Ca^{2+}]_{cyt}$ fluorescence signal **(a)** and quantitative measurement for $[Ca^{2+}]_{cyt}$ levels **(b)** in target leaf 6 at indicated times after application of 100 mM Glu to leaf 1 of *GCaMP3/glr3.3* and *GCaMP3/glr3.6* plants respectively. Data are mean ± SD (n = 3 and 6 for *GCaMP3/glr3.6* and *GCaMP3/glr3.3* in panel b). Scale bars: 2 mm. Source data are provided as a Source Data file.

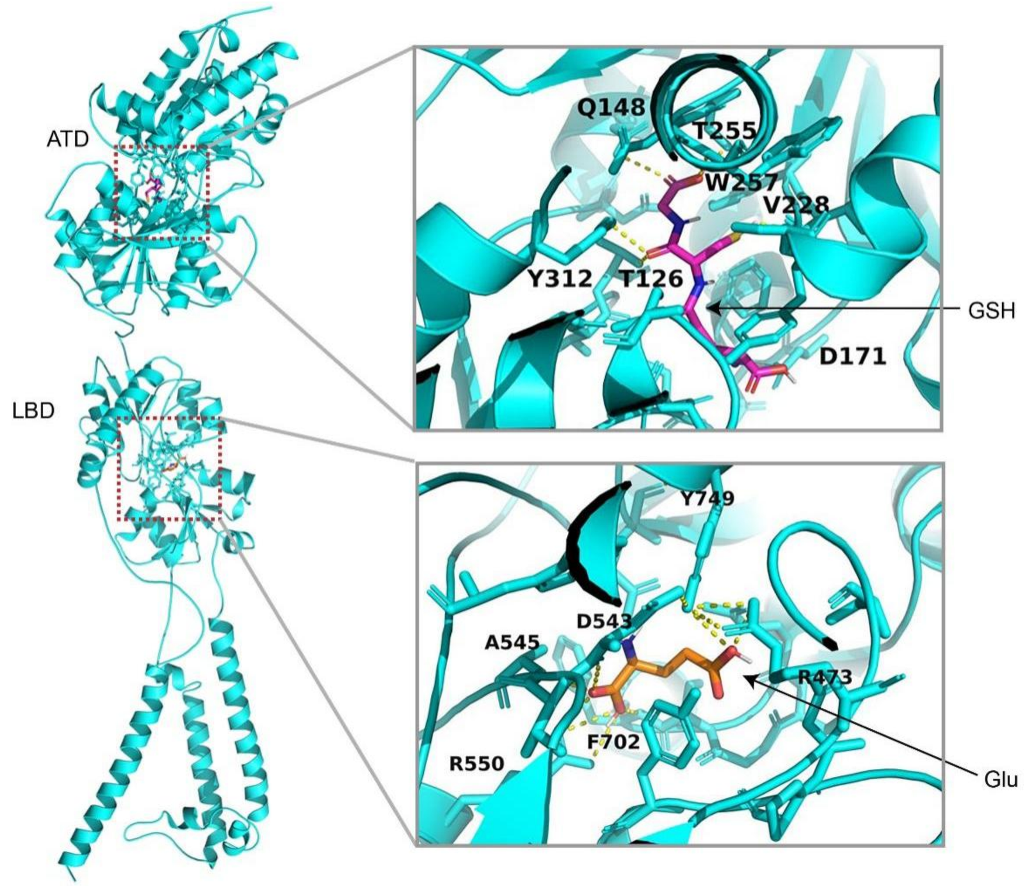

Supplementary Figure 11. Prediction of GSH and Glu binding with GLR3.3 by molecular docking. Structure of AtGLR3.3 with its ligands (right). The GSH binding pocket in ATD (top-right) and Glu binding pocket in LBD (bottom-right) are shown. Ligands and cavity residues within 5 Å of ligand molecules are presented in stick. The polar interactions between ligands and pockets are depicted by yellow dashed lines.

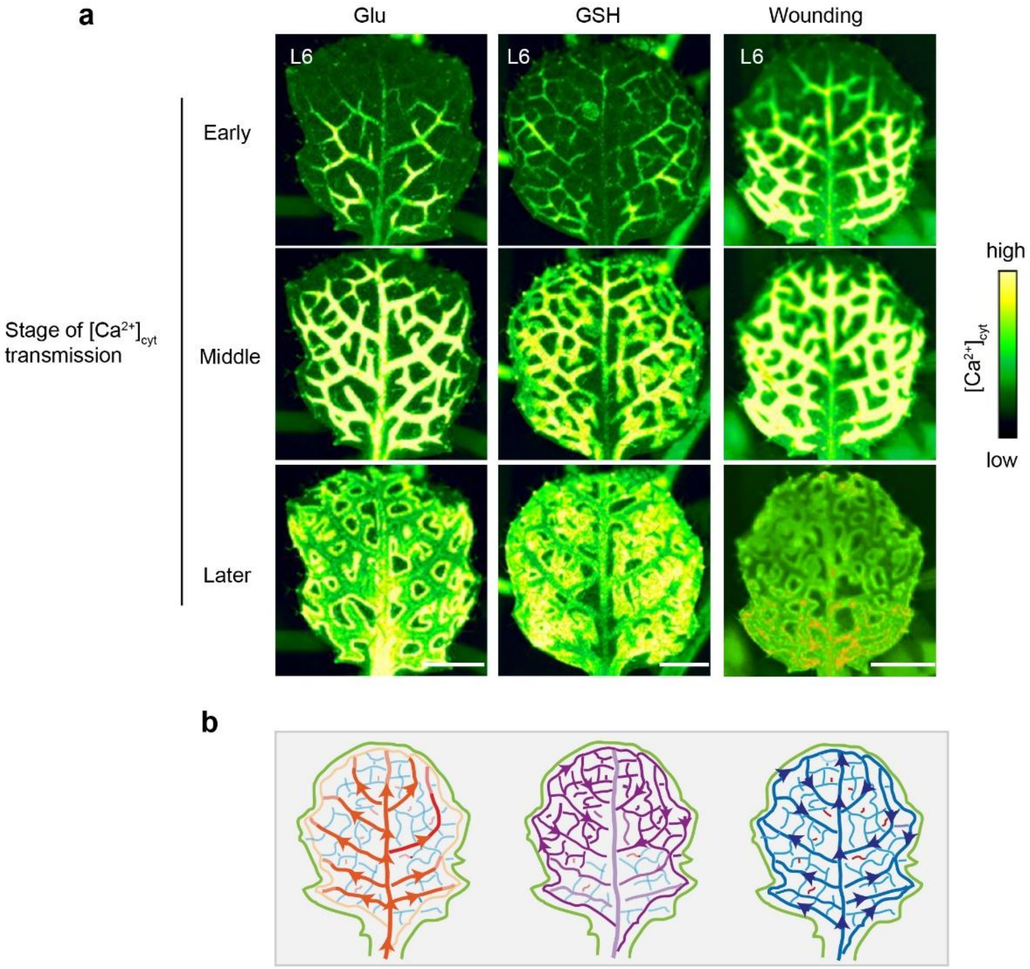

Supplementary Figure 12. Distinct calcium transmission pattern in plant systemic leaves was triggered by GSH and Glu. a The representative images of early, middle, and later stages of GSH-, Glu- and wound-triggered $[Ca^{2+}]_{cyt}$ transmission in leaf 6 (L6) of *GCaMP3* plants. Note that GSH triggers the systemic $[Ca^{2+}]_{cyt}$ propagation in the marginal-to-mid vein direction (refers to Supplementary Movie 6 for details) while Glu induces the mid-to-marginal vein $[Ca^{2+}]_{cyt}$ transmission (refers to Supplementary Movie 7 for details). Wound-indued $[Ca^{2+}]_{cyt}$ transmission contains both marginal-to-mid vein and mid-to-marginal vein directions (refers to Supplementary Movie 8 for details). Scale bars: 1 mm. **b** The schematic diagrams of transmission directions of $[Ca^{2+}]_{cyt}$ triggered by Glu (left), GSH (middle), and wounding (right) in systemic leaves.
