# Supplementary material for: Glutathione triggers leaf-to-leaf, calcium-based plant defense signaling
Source: Nat Commun. 2025 Feb 24;16:1915. doi: 10.1038/s41467-025-57239-1 (PMC11850895; doi:10.1038/s41467-025-57239-1)
Supplement: Supplementary file 2 — Description of Additional Supplementary Files [file 41467_2025_57239_MOESM2_ESM.pdf]

Description of Additional Supplementary files

Supplementary Data 1. List of DEGs in systemic leaf 6 one hour after application of GSH to leaf 1 of WT plants compared to non-stimulated mock control plants.

Supplementary Data 2. GO analysis of DEGs in systemic leaf 6 one hour after application of GSH to leaf 1 compared to non-stimulated mock control plants.

Supplementary Data 3. List of DEGs in systemic leaf 6 one hour after wounding leaf 1 of WT plants compared to uninjured mock control plants.

Supplementary Data 4. List of DEGs in systemic leaf 6 one hour after application of Glu to leaf 1 of WT plants compared to non-stimulated mock control plants.

Supplementary Data 5. Primers used in this study

Supplementary Movie 1. Mechanical wounding (scissors) leaf 1 (L1) in *GCaMP3/pad2-1* mutant plant showing reduced Ca^{2+} increases in leaf 6 (L6).

Supplementary Movie 2. Mechanical wounding (scissors) leaf 1 (L1) in *GCaMP3* plant showing normal Ca^{2+} increases in leaf 6 (L6).

Supplementary Movie 3. Systemic transmission of $[Ca^{2+}]_{cyt}$ elevation in *GCaMP3* plant in response to 100 mM GSH added to the wound of leaf 1.

Supplementary Movie 4. Systemic transmission of $[Ca^{2+}]_{cyt}$ elevation in *GCaMP3/glr3.3* mutant in response to 100 mM GSH added to the wound of leaf 1.

Supplementary Movie 5. Systemic transmission of $[Ca^{2+}]_{cyt}$ elevation in *GCaMP3/glr3.6* mutant in response to 100 mM GSH added to the wound of leaf 1.

Supplementary Movie 6. The $[Ca^{2+}]_{cyt}$ transmission pattern in leaf 6 (L6) in response to 100 mM GSH added to the wound of leaf 1 (L1) in *GCaMP3* plant.

Supplementary Movie 7. The $[Ca^{2+}]_{cyt}$ transmission pattern in leaf 6 (L6) in response

to 100 mM Glu added to the wound of leaf 1 (L1) in *GCaMP3* plant.

Supplementary Movie 8. The $[Ca^{2+}]_{cyt}$ transmission pattern in leaf 6 (L6) in response to mechanical wounding (scissors) leaf 1 (L1) in *GCaMP3* plant.
