# Supplementary material for: Glutathione triggers leaf-to-leaf, calcium-based plant defense signaling
Source: Nat Commun. 2025 Feb 24;16:1915. doi: 10.1038/s41467-025-57239-1 (PMC11850895; doi:10.1038/s41467-025-57239-1)
Supplement: Supplementary file 16 — Reporting Summary [file 41467_2025_57239_MOESM16_ESM.pdf]

nature portfolio

Corresponding author(s): Daoxin Xie, Xiaoyi Shan, Jianbin Yan, Guo-Liang Wang

Last updated by author(s): Jan 8, 2025

Reporting Summary

Nature Portfolio wishes to improve the reproducibility of the work that we publish. This form provides structure for consistency and transparency in reporting. For further information on Nature Portfolio policies, see our Editorial Policies and the Editorial Policy Checklist.

Statistics

For all statistical analyses, confirm that the following items are present in the figure legend, table legend, main text, or Methods section.

n/a	Confirmed	
☐	☒	The exact sample size (n) for each experimental group/condition, given as a discrete number and unit of measurement
☐	☒	A statement on whether measurements were taken from distinct samples or whether the same sample was measured repeatedly
☐	☒	The statistical test(s) used AND whether they are one- or two-sided<br>*Only common tests should be described solely by name; describe more complex techniques in the Methods section.*
☒	☐	A description of all covariates tested
☐	☒	A description of any assumptions or corrections, such as tests of normality and adjustment for multiple comparisons
☐	☒	A full description of the statistical parameters including central tendency (e.g. means) or other basic estimates (e.g. regression coefficient) AND variation (e.g. standard deviation) or associated estimates of uncertainty (e.g. confidence intervals)
☐	☒	For null hypothesis testing, the test statistic (e.g. F, t, r) with confidence intervals, effect sizes, degrees of freedom and P value noted<br>*Give P values as exact values whenever suitable.*
☒	☐	For Bayesian analysis, information on the choice of priors and Markov chain Monte Carlo settings
☒	☐	For hierarchical and complex designs, identification of the appropriate level for tests and full reporting of outcomes
☒	☐	Estimates of effect sizes (e.g. Cohen's d, Pearson's r), indicating how they were calculated

Our web collection on statistics for biologists contains articles on many of the points above.

Software and code

Policy information about availability of computer code

Data collection	1) Transgenic Arabidopsis plants stably expressing GCaMP3 were imaged with a motorized epifluorescence (SZX10, Olympus) stereomicroscope equipped with a 1× objective lens and a C13440 digital camera (ORCA-Flash4.0 V3, Hamamatsu Photonics).<br><br>2) JA and JA-Ile were measured using AB Sciex 4500 QTRAP triple quadrupole mass spectrometer (AB SCIEX, MA, USA) equipped with an ACQUITY UPLC™ BEH C18 column (Waters, Eschborn, Germany) (50 × 2.1 mm, 1.7 µm).<br><br>3) GSH and Glu were measured using a 6500 plus QTrap mass spectrometer (AB SCIEX, USA) coupled with a ACQUITY UPLC H-Class system, equipped with a heated electrospray ionization (HESI) probe.<br><br>4) qRT-PCR was performed using SYBR Select Master Mix on ABi7500 real-time PCR system (Life Technologies).
Data analysis	Fiji (Version 1.52); Graphpad Prism (Version 8.0); Microsoft Excel (2016); SPSS

For manuscripts utilizing custom algorithms or software that are central to the research but not yet described in published literature, software must be made available to editors and reviewers. We strongly encourage code deposition in a community repository (e.g. GitHub). See the Nature Portfolio guidelines for submitting code & software for further information.

Data

Policy information about availability of data

All manuscripts must include a data availability statement. This statement should provide the following information, where applicable:

- Accession codes, unique identifiers, or web links for publicly available datasets
- A description of any restrictions on data availability
- For clinical datasets or third party data, please ensure that the statement adheres to our policy

The RNA-seq data generated in this study has been deposited in the Gene Expression Omnibus (GEO) database at NCBI under accession code GSE249592. All other data supporting the findings of this study are available in the main text or the Supplementary Data. Source data are provided in this paper.

Research involving human participants, their data, or biological material

Policy information about studies with human participants or human data. See also policy information about sex, gender (identity/presentation), and sexual orientation and race, ethnicity and racism.

Reporting on sex and gender	NA
Reporting on race, ethnicity, or other socially relevant groupings	NA
Population characteristics	NA
Recruitment	NA
Ethics oversight	NA

Note that full information on the approval of the study protocol must also be provided in the manuscript.

Field-specific reporting

Please select the one below that is the best fit for your research. If you are not sure, read the appropriate sections before making your selection.

☒ Life sciences ☐ Behavioural & social sciences ☐ Ecological, evolutionary & environmental sciences

For a reference copy of the document with all sections, see nature.com/documents/nr-reporting-summary-flat.pdf

Life sciences study design

All studies must disclose on these points even when the disclosure is negative.

Sample size	No statistical approach was used to predetermine sample size. The sample sizes were determined based on the authors' previous experience with the indicated assays or the relevant literatures.
Data exclusions	No data was excluded from analysis.
Replication	All data at least have three replications.
Randomization	Different genotypes of the plants were randomly positioned in the growth chambers. Plants used were randomly selected from a larger pool of the indicated genotypes growing in identical conditions.
Blinding	Blinding was not employed in the experiments, as the research materials were plants.

Reporting for specific materials, systems and methods

We require information from authors about some types of materials, experimental systems and methods used in many studies. Here, indicate whether each material, system or method listed is relevant to your study. If you are not sure if a list item applies to your research, read the appropriate section before selecting a response.

Materials & experimental systems

n/a	Involved in the study	
	x	Antibodies
x		Eukaryotic cell lines
x		Palaeontology and archaeology
	x	Animals and other organisms
x		Clinical data
x		Dual use research of concern
	x	Plants

Methods

n/a	Involved in the study	
x		ChIP-seq
x		Flow cytometry
x		MRI-based neuroimaging

Antibodies

Antibodies used	Mouse monoclonal anti-Myc, Abmart, Cat#M20002. Dilution used: 1:5000
Validation	The validation of antibody profile can be found in the following link: http://www.ab-mart.com.cn/page.aspx?node=%2059%20&id=%20962

Animals and other research organisms

Policy information about studies involving animals; ARRIVE guidelines recommended for reporting animal research, and Sex and Gender in Research

Laboratory animals	The lower invertebrate Spodoptera exigua was used for insect feeding experiment.
Wild animals	The study did not involve wild animals. The Spodoptera exigua larvae (2nd instar) were purchased from Jiyuan Baiyun Industry Co., Ltd (China).
Reporting on sex	Spodoptera exigua of mixed sex were used for the insect feeding assay. Sex was not considered in the study design, as there is no conclusive evidence indicating that sex influences the feeding habits of Spodoptera exigua.
Field-collected samples	The study did not involve samples collected from the field.
Ethics oversight	No ethical approval or guidance was required, as research involving the lower invertebrate Spodoptera exigua is not subject to ethical approval.

Note that full information on the approval of the study protocol must also be provided in the manuscript.

Dual use research of concern

Policy information about [dual use research of concern]

Hazards

Could the accidental, deliberate or reckless misuse of agents or technologies generated in the work, or the application of information presented in the manuscript, pose a threat to:

No	Yes	
[x]	[ ]	Public health
[x]	[ ]	National security
[x]	[ ]	Crops and/or livestock
[x]	[ ]	Ecosystems
[x]	[ ]	Any other significant area

Experiments of concern

Does the work involve any of these experiments of concern:

No	Yes	
[x]	[ ]	Demonstrate how to render a vaccine ineffective
[x]	[ ]	Confer resistance to therapeutically useful antibiotics or antiviral agents
[x]	[ ]	Enhance the virulence of a pathogen or render a nonpathogen virulent
[x]	[ ]	Increase transmissibility of a pathogen
[x]	[ ]	Alter the host range of a pathogen
[x]	[ ]	Enable evasion of diagnostic/detection modalities
[x]	[ ]	Enable the weaponization of a biological agent or toxin
[x]	[ ]	Any other potentially harmful combination of experiments and agents

Plants

Seed stocks	Seeds used in this study were generated by our and other laboratories, or ordered from NASC.
Novel plant genotypes	The GCaMP3/pad2-1 was generated by genetic crosses using standard procedures. GCaMP3/glr3.3 and GCaMP3/glr3.6 mutants were generated by CRISPR/Cas9 in WT plants overexpressing GCaMP3. To generate the transgenic plant expressing 35S::PAD2-6myc/pad2-1 (PAD2/pad2-1), the coding sequence of PAD2 was cloned into the binary vector pCAMBIA1300, and then transformed into pad2-1.
Authentication	All transgenic lines were confirmed by sequencing and western blot.
